# Application of Acetate as a Substrate for the Production of Value-Added Chemicals in *Escherichia coli*

**DOI:** 10.3390/microorganisms12020309

**Published:** 2024-02-01

**Authors:** Pengfei Gu, Fangfang Li, Zhaosong Huang, Juan Gao

**Affiliations:** 1 School of Biological Science and Technology, University of Jinan, Jinan 250022, China; bio_huangzs@ujn.edu.cn; 2 Yantai Food and Drug Control and Test Center, Yantai 264003, China; loveecoli@163.com

**Keywords:** acetate, valuable chemicals, *E. coli*, metabolism, tolerance

## Abstract

At present, the production of the majority of valuable chemicals is dependent on the microbial fermentation of carbohydrate substrates. However, direct competition is a potential problem for microbial feedstocks that are also used within the food/feed industries. The use of alternative carbon sources, such as acetate, has therefore become a research focus. As a common organic acid, acetate can be generated from lignocellulosic biomass and C1 gases, as well as being a major byproduct in microbial fermentation, especially in the presence of an excess carbon source. As a model microorganism, *Escherichia coli* has been widely applied in the production of valuable chemicals using different carbon sources. Recently, several valuable chemicals (e.g., succinic acid, itaconic acid, isobutanol, and mevalonic acid) have been investigated for synthesis in *E. coli* using acetate as the sole carbon source. In this review, we summarize the acetate metabolic pathway in *E. coli* and recent research into the microbial production of chemical compounds in *E. coli* using acetate as the carbon source. Although microbial synthetic pathways for different compounds have been developed in *E. coli*, the production titer and yield are insufficient for commercial applications. Finally, we discuss the development prospects and challenges of using acetate for microbial fermentation.

## 1. Introduction

With concerns about climate change generated by non-renewable fossil fuels, biofuel production by microbial cell factories using sustainable and cheap substrates has attracted increased attention [1]. The earliest biofuels mainly relied on edible carbohydrates such as glucose, sucrose, and starch. However, the use of edible substrates as biofuels may compete with the human food chain, potentially risking food security [2]. Consequently, the replacement of current edible feedstocks with less expensive and non-edible alternatives is desired.

Acetate is a simple monocarboxylic acid that can be generated from chemical processes, such as methanol carbonylation. In addition, acetate can be obtained in acetogenic bacteria from C1 gases such as CO and CO_2_. The global market for acetic acid has been estimated to reach 19.6 million tons by 2027 [3]. The current market price of acetate is USD350–450/ton, which is a little cheaper than glucose, priced at USD500/ton [3]. Considering that the microbial production of biofuels and other valuable chemicals urgently needs to replace the present edible substrates with more sustainable feedstock, acetate is a promising alternative to be explored for its potential use in microbial biotechnology. 

Among the various strategies to generate acetate, acetate formed from sugars, lignocellulosic biomass, or waste materials is environmentally friendly and economically viable. Considering that feedstocks such as pure sugars from corn or sugar beet compete with the food industry, raw materials such as lignocellulosic biomass, food waste, and gas fermentation, which do not compete with the food or feed industries, seem to be a more sustainable source [2,4]. First, lignocellulosic biomass contains carbohydrates (such as glucose, mannose, galactose, xylose, and arabinose) in the form of cellulose and hemicellulose. Because lignocellulosic biomass exhibits a complex and stable chemical structure, pretreatment processes, such as pyrolysis and saccharification, are often carried out before its utilization as a carbon source for microbial fermentation. In the process of pyrolysis, lignocellulosic biomass is degraded into a carbon-rich solid fraction, an energy-rich liquid fraction, and a non-condensable gaseous fraction under oxygen-free conditions. The energy-rich liquid often contains acetate at a concentration of 5–17% [5]. By contrast, the process of saccharification of lignocellulosic biomass can generate acetate up to 10 g/L, resulting from the acetyl groups in cellulose, hemicellulose, and lignin [6]. Second, as an economically feasible feedstock, food waste is gradually becoming an important part of a cleaner and renewable fuel market [7]. Food waste can be generated during the processes of food distribution, cooking, processing, sale, and storage [8]. In general, there are four stages of the anaerobic digestion of food waste—hydrolysis, fermentation, acetogenesis, and methanogenesis—with acetate being generated during acetogenesis. Third, gases such as CO and CO_2_, which are available from industrial sources, represent another carbon source. Carbon-fixing organisms, such as acetogenic bacteria, can achieve acetate synthesis from C1 gases, a process which has positive benefits on the removal of greenhouse gases. Up to 51 g/L of acetate was reportedly obtained from *Acetobacterium woodii* DSM 030 using a CO_2_/H_2_ gas combination as a substrate [9].

In nature, only a few bacteria can naturally assimilate acetate. Among them, *Escherichia coli*, a model microorganism with the advantages of fast growth in cheap media and simple genetic manipulation protocols, has been widely used in the microbial fermentation of different valuable chemicals [10,11]. As a result, microbial production from acetate using *E. coli* as a chassis has become a promising research area. Accordingly, this review focuses on the potential application of acetate as a substrate for the production of valuable chemicals in *E. coli*. The acetate assimilation pathway in *E. coli* is characterized in this study as well as acetate transport and the tolerance of *E. coli* for acetate, including the genes and mechanisms involved. Then, the recent application of acetate in the microbial production of various valuable chemicals, such as succinic acid and isobutanol, is introduced. Finally, the benefits and challenges facing acetate utilization for microbial production in *E. coli* are analyzed. This review provides an overview and a useful reference for acetate utilization in *E. coli*.

## 2. Metabolism of Acetate in *E. coli*

**Figure 1:**
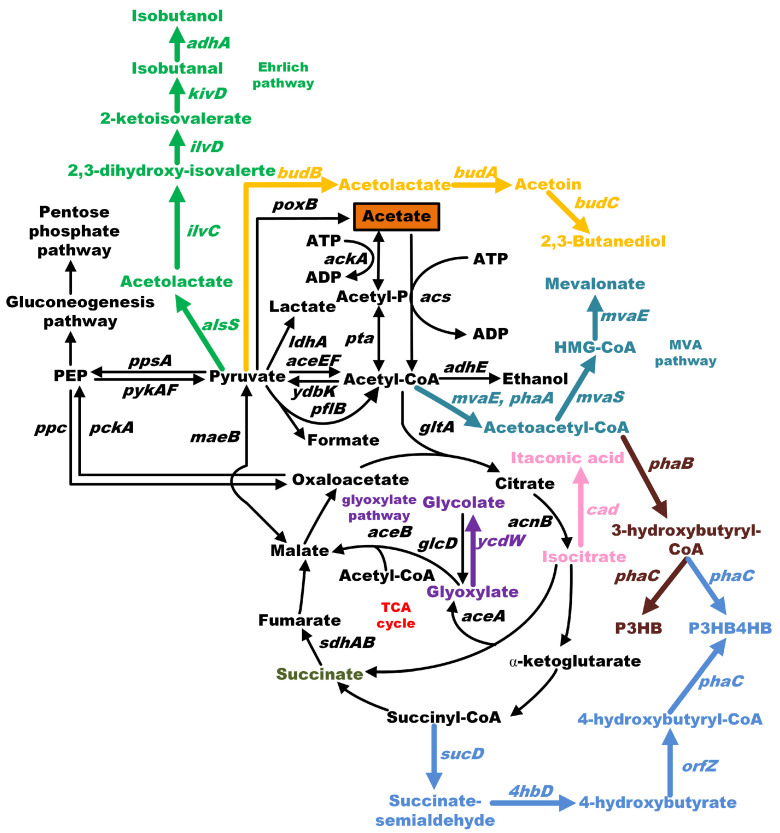
Using acetate as a substrate for the production of value-added chemicals in *E. coli*. The biosynthetic pathways of isobutanol, 2,3-butanediol, mevalonate, itaconic acid, poly-3-hydroxybutyrate (P3HB), succinate, glycolate, and poly(3-hydroxybutyrate-co-4-hydroxybutyrate) (P3HB4HB) are indicated in grass green, yellow, cyan, pink, brown, olive green, purple, and blue, respectively.

In *E. coli*, acetate can enter the central metabolic pathway via an important intermediate, acetyl-CoA (Figure 1) [12]. Subsequently, acetyl-CoA can be transformed into other carbon compounds via the tricarboxylic acid (TCA) cycle, the gluconeogenesis pathway, and the glyoxylate cycle. The conversion of acetate into acetyl-CoA can be achieved by acetate kinase AckA and phosphate acetyltransferase Pta. In addition, acetyl-CoA synthetase encoded by the *acs* gene can catalyze acetate into acetyl-CoA [13]. However, the reaction catalyzed by the AckA-Pta pathway is reversible, whereas the acetyl-CoA synthetase pathway is irreversible due to acetyl-AMP acting as an intermediate. Both of these pathways require one ATP to transform acetate into acetyl-CoA, but the Acs pathway is more energy-consuming due to the pyrophosphate (PPi) obtained when acetyl-AMP is further transformed into two Pi [14].

The AckA-Pta pathway is also involved in acetate secretion when *E. coli* is cultivated with a carbon source such as glucose. One mole of ATP could be generated by substrate-level phosphorylation in the AckA-Pta pathway, which is critical for *E. coli* growth, especially under anaerobic conditions [15]. In addition, acetate secretion by *E. coli* can be accomplished by pyruvate oxidase, encoded by *poxB*. To alleviate or weaken the negative impact of acetate on strain growth, three genes (i.e., *ackA*, *pta*, and *poxB*) are often deleted or suppressed to decrease acetate secretion and redirect more of the carbon flow toward the biosynthesis of target compounds [16,17,18]. 

Interestingly, the functions of the AckA-Pta pathway and the Acs pathway are different when acetate is selected as the sole carbon source. Wild-type *E. coli* grows well at an acetate range of 2.5–50 mM (~0.15–3 g/L) [2]. By contrast, mutant *E. coli* with deletion of the *acs* gene exhibits poor growth at acetate concentrations below 10 mM, while mutant strains with *pta*-*ackA* deficiency grow poorly when the acetate concentration is above 25 mM. In addition, no obvious growth with acetate can be observed for the mutant *E. coli* strain with the deletion of both *acs* and *pta-ackA*. This phenomenon suggests that both the Pta-AckA and Acs pathway are vital for *E. coli* growth at different acetate concentrations.

The glyoxylate cycle is also important for *E. coli* growth when acetate is employed as the sole carbon source. This pathway begins with the splitting of isocitrate into glyoxylate and succinate. Then, glyoxylate and acetyl-CoA can generate malate, which is directed into the TCA cycle. Therefore, the glyoxylate pathway can generate one C4 compound malate from the two C2 compound acetyl-CoA [19]. Malate is then oxidized to fumarate and oxaloacetate in turn, which can be used for the synthesis of higher carbon compounds and as an energy supply. In *E. coli*, enzymes involved in the glyoxylate cycle are encoded by the *aceBAK* operon under the regulation of isocitrate lyase regulator IclR. Accordingly, direct deletion or repression is often applied for improving acetate utilization and the accumulation of TCA cycle-derived compounds, such as L-threonine [20] and succinate [21].

## 3. Acetate Transport in *E. coli*

Acetate can be transported into wild-type *E. coli* by means of passive or active transport, although active transport predominates. The passive transport of acetate is dependent on the concentration gradient between the intracellular and extracellular acetate concentrations. The undissociated form of acetic acid can enter into *E. coli* via pores and/or facilitator proteins in the cell membrane. After entering into the cell, the acetic acid rapidly dissociates to an acetate anion and a proton [2]. By contrast, acetate can be transported by a sodium:solute symporter or a H^+^/monocarboxylic acid symporter. In *E. coli*, the sodium:solute symporter is also responsible for the transport of sugars, amino acids, and nucleosides [22].

In *E. coli*, the *actP* gene is located adjacent to the *acs* gene, which encodes acetate permease [23]. ActP can transport glycolate and propionate. When glucose has been consumed and an *E. coli* strain enters the early stationary phase, the expression of *actP* is increased [24]. Apart from ActP, carboxylic acid transporter YaaH can transport acetate in *E. coli*. ActP exhibits a high specificity for acetic acid and succinic acid, indicated by affinity constants of 1.24 ± 0.13 mM for acetic acid and 1.18 ± 0.10 mM for succinic acid at pH 6.0.

## 4. Acetate Tolerance of *E. coli*

In batch or fed-batch fermentation, the normal growth of wild-type *E. coli* is severely impaired when the acetate concentration exceeds 5 g/L. It has been observed that acetate can prolong the lag phase and reduce biomass accumulation [25]. Accordingly, the cultivation of *E. coli* with acetate at a high cell density is often limited. The cellular damage caused by acetate is probably due to the co-existence of non-dissociated and dissociated forms of acetate [26]. The non-dissociated form has been reported to be more toxic than the dissociated form of acetate for microbial growth [27]. 

Apart from decreasing acetate secretion, engineered *E. coli* strains for enhancing acetate tolerance may be another effective strategy for the microbial production of valuable chemicals. A recombinant *E. coli* strain KO11 containing the *pdc* and *adh* genes of *Zymomonas mobilis* was tested for acetate tolerance. When a Luria Broth medium with 2.0–12.0 g/L acetate was employed, neither the yield nor the productivity of ethanol was exhibited for the *E. coli* strain KO11 [25]. In another report, the heterologous expression of DcHsp17.7 from carrot was shown to overcome the growth inhibition effect of acetate for *E. coli* [28]. As the global regulator cAMP receptor protein (CRP) is responsible for regulating the expression of over 400 genes, error-prone PCR was performed for CRP to select mutant *E. coli* strains with an increased acetate tolerance [29]. In the presence of 15 g/L of sodium acetate, mutant strain A2 (D138Y) exhibited a higher growth rate than the control (0.083 h^−1^ vs. 0.016 h^−1^). Using a real-time PCR analysis, the overexpression of *uxaB* encoding tagaturonate reductase was found to be crucial for *E. coli* acetate sensitivity. In addition, RpoS, the stress sigma factor of *E. coli*, plays an important role in acetate tolerance under a low but non-lethal pH or upon entering into the stationary phase [30]. 

Laboratory metabolic evolution could also be applied to increase the acetate tolerance of *E. coli*. From wild-type *E. coli* MG1655, an acetate-tolerant *E. coli* mutant strain MS04 was obtained [26]. By genome sequencing, a 27.3 kb deletion comprising enzymes for nitrate respiration, repair of alkylated DNA, and the synthesis of porin C, cytochrome C, thiamine, and colonic acid was identified on the chromosome of the mutant strain. These results indicated that the acetate tolerance of *E. coli* may not be dependent on a single pathway but, instead, that a combination of various mechanisms may be involved in overcoming the growth inhibition of acetate [31].

## 5. Using Acetate as a Substrate for the Production of Value-Added Chemicals in *E. coli*

**Figure 2:**
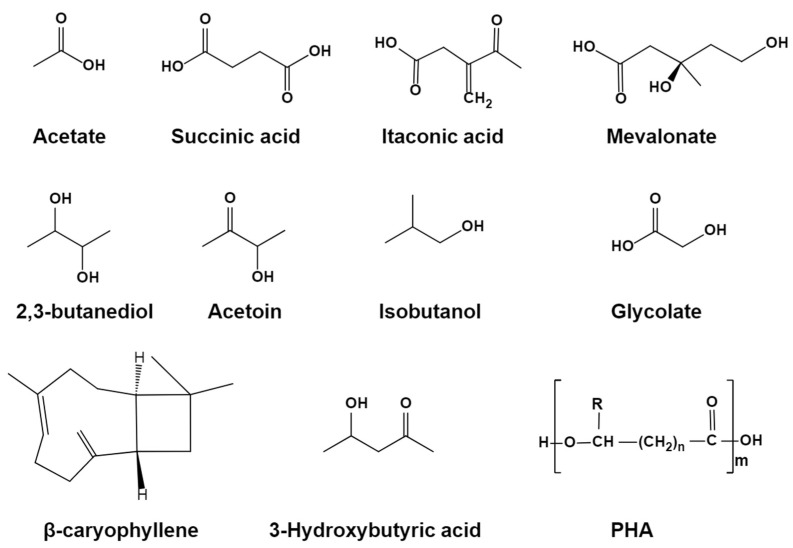
Chemical structures of acetate and chemicals synthesized from acetate in *E. coli*.

As a key metabolite in *E. coli*, acetyl-CoA can be easily obtained from acetate. As a result, chemical compounds derived from acetyl-CoA are good candidates for use with acetate as the sole carbon source. To date, the production of several chemical compounds has been investigated (Figure 2 and Table 1). 

### 5.1. Succinic Acid

Succinic acid is an important four-carbon platform compound that is widely applied in the food, pharmaceutical, and chemical industries [32]. Succinic acid is also one of the catalogues of top 12 building-block chemicals published by the US Department of Energy. Succinate is also employed as a precursor to some biodegradable polyesters [33,34]. In 2016, Li et al. engineered a recombinant *E. coli* strain, MG03, for the use of acetate as a substrate [35]. To increase the succinate titer, several metabolic engineering strategies were implemented, such as blocking the TCA cycle, redirecting the gluconeogenesis pathway, and activating the glyoxylate shunt. The final strain, MG03(pTrc99a-gltA), could accumulate 16.45 mM succinate with a yield of 0.46 mol/mol. Similarly, our laboratory has developed a recombinant *E. coli* strain, WCY-7, using similar methods, with the additional overexpression of the *acs* gene encoding acetyl-CoA synthetase and the *acnB* gene encoding aconitate hydratase on a plasmid. After 48 h of batch fermentation, WCY-7 could accumulate 11.23 mM succinate from 50 mM sodium acetate [21]. On the basis of wild-type *E. coli* strain BW25113, Huang et al. constructed a succinic acid-producing *E. coli* mutant that uses acetate as a substrate, with weakened OAA decarboxylation, fine-tuning of the TCA cycle, and increased acetate utilization and ATP production [36]. This strain could accumulate 30.9 mM succinate with a yield of 0.50 mol/mol, with the succinate titer further increasing to 194 mM in resting-cell experiments. 

### 5.2. Itaconic Acid

As a C5 unsaturated dicarboxylic acid, itaconic acid can be used as a building block for plastics and latex [37]. Similar to succinic acid, itaconic acid was also selected as one of the top 12 bio-based platform chemicals in 2004. Itaconic acid can be synthesized directly from isocitrate, an intermediate of the TCA cycle. Accordingly, this compound can also be investigated for its possible production from acetate. To overcome cell inhibition by acetate, Noh et al. screened and identified an acetate-tolerant strain that could grow under 10 g/L of acetate. After overexpressing the *cis*-aconitate decarboxylase, the carbon flux was directed into acetate utilization, and the glyoxylate shunt was increased, with the final strain, WCIAG4, being able to produce 3.57 g/L itaconic acid with a yield of 16.1%, along with the rapid assimilation of acetate [38]. 

### 5.3. Mevalonate (MA)

Mevalonate is a key organic compound that can be used as a precursor for the synthesis of steroids and terpenes. Mevalonate can also be transformed into artemisinin, taxol, and tanshinone for disease treatment as well as carotene and lycopene for use as food additives [39,40,41]. In nature, mevalonate can be synthesized via the mevalonate pathway in most eukaryotes and higher plants. A complete biosynthetic pathway for mevalonate using sugars as substrates was constructed in recombinant *E. coli* [42,43]. In 2018, Xu et al. constructed a recombinant *E. coli* strain with acetate as the sole carbon source for the production of mevalonate [44]. The final strain, XU143, was obtained by overexpressing *acs* and *Enterococcus faecalis*-derived *mvaE* and *mvaS* encoding acetyl-CoA acetyltransferase and HMG-CoA synthase, respectively, in *E. coli* BL21(DE3). In fed-batch fermentation, mevalonate production reached 1.06 g/L with a yield of 0.30 g/g acetate for strain XU143. 

### 5.4. Isobutanol

As a representative biofuel, isobutanol is a potential alternative to fossil fuels that is considered relatively low risk in terms of pollution. In addition, isobutanol can be used as a precursor for several valuable chemicals, such as polymers, paint, and plastics [45]. As isobutanol can be derived from pyruvate, to expand the available substrates, acetate was also investigated for isobutanol production in *E. coli*. On the basis of wild-type strain MG1655(DE3), Song et al. constructed recombinant *E. coli* strain HM501::MAP via the engineering of the acetate assimilation pathway, the by-products formation pathway, the TCA cycle, and the isobutanol synthetic pathway [46]. Strain HM501::MAP could synthesize approximately 125 mg/L isobutanol after 120 h of cultivation. Recently, our laboratory also achieved isobutanol production from acetate in *E. coli*. Unlike strain HM501::MAP, we investigated the function of the pyruvate-ferredoxin oxidoreductase YdbK in the production of isobutanol [47]. After blocking the competitive pathway and overexpressing isobutanol synthetic pathway genes, the final strain, WY002, could produce 157.05 mg/L isobutanol from 50 mM acetate, representing the highest isobutanol titer using acetate as the single carbon source in batch cultivation.

### 5.5. 2,3-Butanediol and Acetoin

2,3-butanediol is a promising platform chemical that can be used as a food additive and an anti-freezing agent [48]. Acetoin can be used as a flavor enhancer [49]. In 2020, Novak et al. constructed a complete pathway of acetoin and 2,3-butanediol using acetate as substrate in *E. coli* [50]. By deletion of the mixed-acid fermentation pathways, the *E. coli* strain WΔ4 could produce 0.62 g/L of 2,3-butanediol and acetoin. In addition, the authors found that acetoin and 2,3-butanediol production from acetate was mediated by the availability of aspartate. After careful optimization of the culture media, the total titer of 2,3-butanediol and acetoin from *E. coli* WΔ4 was increased to 1.16 ± 0.02 g/L, with a yield of 0.067 ± 0.02 g/g acetate. 

### 5.6. Glycolate

Glycolate is the simplest member of the α-hydroxy acids and is applied in the textile, oil, and gas industries [51]. Glycolate can also act as a monomer with lactate for synthesizing biodegradable and biocompatible materials [52]. As glycolate can be obtained from glyoxylate via glyoxylate/hydroxypyruvate reductase, this chemical can also be explored for production in *E. coli* from acetate. In 2019, Li et al. constructed a recombinant *E. coli* strain by engineering the native glyoxylate cycle, which involved deleting malate synthase, glyoxylate carboligase, and glycolate oxidase [53]. The final *E. coli* strain, K12 ΔA4 (pGAx4/pEn-gltA-pta-ackA), could produce 2.75 g/L glycolate in a 48 h batch fermentation. 

### 5.7. β-Caryophyllene

β-caryophyllene is a common sesquiterpene that is abundant in plants. β-caryophyllene is a component in next-generation aircraft fuels [54]. Although microbial fermentation of β-caryophyllene has been developed, acetic acid generated under aerobic conditions often interferes with normal strain growth and limits β-caryophyllene production. Accordingly, Yang et al. reconstructed the β-caryophyllene biosynthetic pathway directly from acetic acid in *E. coli* [55]. The acetyl-CoA synthases from three different sources were introduced into *E. coli* to screen for chassis cells with higher acetate utilization efficiencies. In addition, the complete biosynthetic pathway of β-caryophyllene from acetate was constructed, and the heterologous MVA pathway was also introduced into the recombinant *E. coli* strain. The recombinant *E. coli* strain YJM67 could achieve 1.05 g/L of β-caryophyllene with a conversion efficiency of 2.1%. 

### 5.8. 3-Hydroxybutyric Acid and Polyhydroxyalkanoates

3-hydroxybutyric acid (3HB), a monomer for poly-(3-hydroxybutyric acid) (PHB), represents a popular biodegradable alternative to traditional petrochemical-based plastics [56]. In addition, 3HB can be used for the production of vitamins and antibiotics. By overexpressing β-ketothiolase (*phaA*) and acetoacetyl-CoA reductase (*phaB*) from *Ralstonia eutropha* and propionyl-CoA transferases (*pct*) from *Clostridium beijerinckii* 8052, a 3HB biosynthesis pathway was successfully constructed in *E. coli.* The final strain, FP06, could produce 6.86 g/L of 3HB using acetate as the sole carbon source [57]. In addition, when syngas-derived acetate was used with this strain, 3HB titer was further increased to 1.02 g/L, with a yield of 0.26 g/g acetate [57]. In 2018, Chen et al. constructed a recombinant *E. coli* to synthesize poly-3-hydroxybutyrate (P3HB) and poly(3-hydroxybutyrate-co-4-hydroxybutyrate) (P3HB4HB) from acetate [58]. The recombinant strain with overexpressed phosphotransacetylase/acetate kinase and the P3HB synthesis operon could produce 1.27 g/L P3HB with 5 g/L acetate in batch fermentation. Further introducing succinate semialdehyde dehydrogenase, 4-hydroxybutyrate dehydrogenase, and CoA transferase resulted in the accumulation of 1.71 g/L P3HB4HB. 

### 5.9. Sweet Protein

Considering that acetate can be employed as a cheap carbon source and that *E. coli* is the most commonly used prokaryotic protein-expressing host (*E. coli* strain BL21(DE3) in particular), the production of recombinant proteins from acetate has also been investigated. MNEI, a single-chain derivative of the sweet plant protein monellin was selected as a target. The MNEI obtained from acetate could achieve a purity higher than 99%. Oxygenation and pH levels were shown to be vital for protein production from acetate [59].

**Table 1:** Fermentation of high-value products using acetate as the sole carbon source in *E. coli*.

Strain	Relevant Characteristics	Products	Titer	Acetate Conversion Rate	Fermentation Conditions	References
MG03(pTrc99a-gltA)	MG1655 (Δ*sdhAB*Δ*iclR*Δ*maeB*Δ*poxB*) with overexpressed *gltA*	succinate	16.45 mM	0.46 mol/mol	Batch fermentation in 25 mL flask at 37 °C and 220 rpm, SMAC medium	[35]
HB03(pTrc99a-gltA, pBAD33-Trc-fdh)	MG1655(Δ*sdhAB*Δ*iclR*Δ*maeB*Δ*pckA*Ptrc-m2-*ackA*-*pta*) with overexpressed *fdh* from *Candida boidinii*	succinate	30.9 mM	0.50 mol/mol	Batch fermentation in 250 mL flask containing 50 mL of NH_4_Cl-free M9 medium at 37 °C and 220 rpm	[36]
WCY-7	MG1655 (Δ*iclR*Δ*sdhAB*Δ*maeB*) with overexpressed *acs*, *gltA* and *acnB*	succinate	11.23 mM	0.22 mol/mol	Batch fermentation in 300 mL flask containing SMAC medium at 37 °C and 250 rpm	[21]
WCIAG4	Acetate-tolerant *E. coli* W with deletion of *iclR* and overexpression of *cad*, *acs*, *gltA*, and *aceA*	itaconic acid	3.57 g/L	0.161 g/g	Fed-batch in 5 L bioreactor containing 1.5 L of modified minimal acetate medium at 30 °C and 500 rpm	[38]
XU143	BL21(DE3) with overexpressed *acs* and *Enterococcus faecalis*-derived *mvaE* and *mvaS*	mevalonate	1.06 g/L	0.30 g/g	Fed-batch in 5 L bioreactor containing 2 L of minimal medium	[44]
HM501::MAP	MG1655(DE3) (Δ*frdA*Δ*pta*Δ*ldhA*Δ*adhE*) with overexpressed *alsS*, *kivD*, *ilvC*, *ilvD*, *yqhD*, *acs*, *pckA*, and *maeB*	isobutanol	0.125 g/L	0.042 g/g	Batch fermentation in 250 mL screw-cap flask containing 30 mL of M9 minimal medium at 30 °C and 200 rpm	[46]
WY002	BW25113 (Δ*pflB*Δ*poxB*Δ*adhE*Δ*ldhA*) with overexpressed *adhA*, *kivD*, *alsS*, *pckA*, *maeB*, *alsS*, *ilvC*, *ilvD*, *acs*, *pntA*, and *yfjB*	isobutanol	0.157 g/L	0.052 g/g	Batch fermentation in 300 mL flask containing 50 mL of medium at 30 °C and 200 rpm	[47]
WΔ4	*E. coli* W (Δ*ldhA*Δ*adhE*Δ*pta* Δ*frdA*) with overexpressed *budA*, *budB*, and *budC* from *Enterobacter cloacae* subsp. *dissolvens*	2,3-butanediol and acetoin	1.16 g/L	0.09 g/g	Pulsed fed-batch fermentation in a bioreactor with a working volume of 200 mL at 30 °C and 800 rpm	[50]
K12 ΔA4 (pGAx4/pEn-gltA-pta-ackA)	MG1655(DE3) (Δ*endA* Δ*recA*Δ*xylB*Δ*gcl*Δ*aceB*Δ*glcB*Δ*glcD*) with overexpressed ycd, *aceA*, *aceK*, *gltA*, *pta*, and *ackA*	glycolate	2.75 g/L	0.58 g/g	Batch fermentation in 500 mL shake flasks containing 50 mL minimal medium at 37 °C and 200 rpm	[53]
YJM67	BL21(DE3) with overexpressed QHS1 from *Artemisia annua*, *mvaE* and *mvaS* from *Enterococcus faecalis*, GPPS2 from *Abies grandis*, ACSAP from *Acetobacter pasteurianus*, nphT7 from *Streptomyces* sp. strain CL190, and *ERG12*, *ERG8*, *ERG19*, and *IDI1* from *Saccharomyces cerevisiae*	β-caryophyllene	1.05 g/L	0.021 g/g	Fed-batch in 5 L fermenter containing 2 L of M9 minimal medium at 30 °C	[55]
FP06	BW25113 with overexpressed *phaA* and *phaB* from *R. eutropha*, and *pct4543* from *C. beijerinckii* 8052	(R)-3-hydroxybutyric acid	0.86 g/L	0.27 g/g	Batch fermentation in 500 mL shake flasks containing 50 mL minimal medium at 37 °C and 200 rpm	[57]
JM109 (pBHR68 + pMCS-acs)	JM109 containing *phaCAB* and *acs*	poly-3-hydroxybutyrate	1.27 g/L	0.254 g/g	Batch fermentation in 250 mL shake flasks containing 50 mL SMAC media at 37 °C and 220 rpm	[58]

## 6. Challenges and Future Perspectives

As a low-cost substrate, acetate has been employed for the synthesis of various chemicals in *E. coli*. However, present studies on the economical production of chemicals from acetate are still in their early stages. To further expand the application of acetate as a substrate in *E. coli*, a few challenges need to be overcome. First, acetate is toxic for *E. coli*, especially when the acetate concentration exceeds 5 g/L. In addition, acetate can extend the lag phase and interfere with biomass accumulation in *E. coli*. Accordingly, acetate cannot be added all at once and should be supplemented gradually during fed-batch fermentation. However, increasing the acetate tolerance of *E. coli* by means of adaptive evolution is an optional strategy. Second, acetate represents one of the oxidized byproducts of *E. coli*. When acetate is employed for the production of highly reduced chemicals, the production titer and yield are often limited. In addition, the energy content of acetate is relatively lower than that of the commonly used substrates glucose and sucrose. As a result, the production of high carbon-numbered products is difficult using acetate in *E. coli*. To address this problem, careful engineering and balance of the acetate assimilation pathway, the central metabolic pathway, and the products synthesis pathway are crucial. In *E. coli*, the TCA cycle and the electron transport chain are mainly responsible for energy generation, while the acetyl-CoA directly generated from acetate is a key node of the TCA cycle and the biosynthesis of crucial compounds for *E. coli*. Accordingly, carbon flow distribution at the acetyl-CoA node between energy generation and the synthesis of compounds is pivotal, but this is still highly challenging to achieve at present. Third, the acquisition of cheap acetate is still an issue to be addressed. Acetate synthesis from C1 gases, such as CO and CO_2_, is the most attractive option because of its potential benefits regarding climate change. However, commercialized strategies for the biological conversion of C1 gases to acetate are yet to be developed because of difficulties in strain engineering, fermentation operation, and downstream processing. Fourth, to facilitate the use of raw acetate obtained from plant biomass, waste food, or C1 gases, the acetate concentration in a liquid should be improved compared with other raw carbon sources; this is vital for the sustained and commercial microbial production of valuable chemicals from acetate. Techno-economic analyses are a widely used tool for analyzing the technical and economic performance of a process in various industries, including chemical production, while life cycle assessments are a methodology for quantifying a wide range of environmental impacts [60,61]. To provide a reference for future commercial applications, techno-economic analyses and life cycle assessments are necessary for acetate-based microbial fermentation. Further extensive investigations to solve these issues should be carried out.

## 7. Conclusions

Acetate represents a potential feedstock for biotechnological processes because of its low cost. In this review, the metabolic pathway for acetate in *E. coli* was described, followed by recent developments in using acetate as a carbon feedstock for the microbial production of chemical compounds such as succinic acid, itaconic acid, and mevalonic acid in *E. coli*. However, studies on the economical production of chemicals from acetate are still at a preliminary stage. Further studies to improve the production titer, productivity, and yield obtained when using acetate to similar levels to those obtained when using popular carbon sources such as glucose, sucrose, and glycerol are desired for potential commercial applications. Acetate warrants further consideration as a potential carbon source for microbial cell factories.

## Data Availability

Not applicable.
